# Data on microhardness and structural analysis of friction stir spot welded lap joints of AA5083-H116

**DOI:** 10.1016/j.dib.2020.106585

**Published:** 2020-11-30

**Authors:** Esther T. Akinlabi, Ayuba S. Osinubi, Nkosinathi Madushele, Stephen A. Akinlabi, Omolayo M. Ikumapayi

**Affiliations:** a Pan African University for Life and Earth Sciences Institute (PAULESI), Ibadan, Nigeria; b Department of Mechanical Engineering Science, University of Johannesburg, Johannesburg, South Africa; c Department of Mechanical Engineering, Faculty of Engineering and Technology, Butterworth Campus, Walter Sisulu University, South Africa; d Department of Mechanical and Mechatronics Engineering, Afe Babalola University, Ado Ekiti, Nigeria

**Keywords:** AA5083-H116, Friction-stir-spot-welding, Microhardness, Structural-integrity, XRD

## Abstract

Friction stir spot welding (FSSW) was established to compete reasonably with the reverting, bolting, adhesive bonding as well as resistance spot welding (RSW) which have been used in the past for lap joining in automobile, aerospace, marine, railways, defence and shipbuilding industries. The use of these ancient and conventional joining techniques had led to increasing material cost, installation labour, and additional weight in the aircraft, shipbuilding, and other areas of applications. All these are disadvantages that can be overcome using FSSW. This research work carried out friction stir spot welding on 5058-H116 aluminium alloy by employing rotational speed in the step of 300 rpm ranges from 600 rpm to 1200 rpm with a no travel speed. It was noted that the dwell times were in the step of 5 s varying from 5 s to 15 s while the tool plunge rate was maintained at 30 mm/min. In this dataset, a cylindrical tapered rotating H13 Hot-working steel tool was used with a probe length of 5 mm and probe diameter of 6 mm, it has a shoulder diameter of 18 mm. The tool penetration depth (plunge) was maintained at 0.2 mm and the tool tilt angle at 2°. Structural integrity was carried out using Rigaku ultima IV multifunctional X-ray diffractometer (XRD) with a scan voltage of 40 kV and scan current of 30 mA. This was used to determine crystallite sizes, peak intensity, d-spacing, full width at half maximum intensity (FWHM) of the diffraction peak. TH713 digital microhardness equipment with diamond indenter was used for microhardness data acquisition following ASTM E92–82 standard test. The average Vickers hardness data values at different zones of the spot-welds were captured and presented.

## Specifications Table

**Table:** 

Subject	Material Science
Specific subject area	Friction Stir spot welding
Type of data	Table Graph Figure
How data were acquired	Structural analysis dataset in the tables was acquired from the experiment carried out on the fabricated aluminium metal alloy AA5083-H116 using X-Ray Diffraction (XRD) while Microhardness were acquired using Vickers hardness through diamond indenter following ASTM E92–82 standard. The chemical compositions of the base material and the tool were acquired through optical emission spectrophotometer.
Data format	Raw Analysed
Parameters for data collection	The welding parameters used in the collection of these data are rotational speed of 600 rpm, 900 rpm and 1200 rpm using welding time of 5 s, 10 s and 15 s, the plunge depth was 0.2 mm and the tilt angle used was 2° while the plunge rate was maintained at 30 mm/min. There was no travel speed used since there is no movement of the tool during the welding.
Description of data collection	Microhardness was experimented on TH713 digital microhardness equipment using diamond indenter based on ASTM E92–82 standard test method with the use of 100 g at 15 s dwell time on each sample. The structural integrity and crystallite sizes were carried out using Rigaku Ultima IV multifunctional x-ray following E112–12 standard test.
Data source location	Friction stir spot welding experiment was carried out at the: Institution: Indian Institute of Technology City/Town/Region: Kharagpur, West Bengal 721,302 Country: India The characterization of the acquired data was conducted at the: Institution: University of Johannesburg City/Town/Region: Johannesburg, Gauteng Country: South Africa
Data accessibility	With the article

## Value of the Data

These data are useful for the determination of microhardness of fabricated AA5083-H116 at different welded zones, the associated crystallite size and peak intensity of the structural materials.

The parameters used for the friction stir spot welding process were carefully selected and optimized which will serve as a guide for future researchers and end-users on the best parameters to work with while working on similar materials.

The chemical compositions of AA5083-H116 and that of non-consumable, high strength AISI H13 chromium hot-work steel tool were determined, and they will be of valuable data for future researchers using these materials.

The microhardness and structural analysis data will be of valuable data for marine industry where AA5083-H16 is prominently used for the joining of ships and vessels.

## 2 Data Description

A commercially available cold-rolled aluminium alloy 5083-H116 plates were the materials used for this friction stir spot welding dataset experiment. The plates of AA5083-H116 were procured from Metal Centre, Johannesburg, South Africa with a dimension of 600 mm X 300 mm X 4 mm which were later cut into the required dimensions (200 mm X 30 mm X 4 mm). The chemical compositions of the procured parent material AA5083-H116 as displayed in Table 1 were determined using optical emission spectrophotometer which was found to conform to the material safety data sheet (MSDS) of the supplier and the standard of AA5083-H116 as reported by Tamasgavabari et al. [1], [2], [3], [4] and conform to the range of the standard datasheet of AA5083 [5]. It was further revealed from MSDS that the parent material AA5083-H116 contains the following properties: ultimate tensile strength of 317 MPa, Shear strength of 109 MPa, tensile yield strength of 228 MPa, shear modulus of 26.4 GPa, modulus of elasticity of 70.3 GPa, Vickers hardness of 96, elongation at break of 16% and poison's ratio of 0.33. Table 2 presents the process parameters used for the fabrication of aluminium alloy samples used for data acquisition. Tables 3 presents the chemical composition of AISI H13 steel tool, while Table 4 presents the design parameters used for the fabrication of AISI H13 steel tool. Table 5 presents XRD structural analysis measurement conditions while Tables 6–9 present XRD peaks list for the spot-welds AA5083-H116 at 600 rpm, 900 rpm and 1200 rpm respectively with their raw data in the attached Supplementary Materials (Raw data for Tables 6–8 respectively). Lastly, Table 10 presents the average Vickers hardness data values at different welding zones. In the same vein, Fig. 4 illustrates the XRD pattern for base metal and the spot-welds samples of AA5083-H116, the raw data for plotting each peak intensity can be found in the attached Supplementary Materials (“Raw data for Fig. 4″). Also, Figs. 5 and 6 present microhardness data values for all the fabricated spot-welds samples and the average microhardness data values at different zones of the spot-welds. The raw data for plotting the microhardness profile in Fig. 5 can be found in the attached Supplementary Material (“Raw data for Fig. 5″).

**Table 1:** Chemical composition of AA5083-H116.

**Element**	Mn	Cr	Fe	Mg	Cu	Zr	Ti	Si	Zn	Al
**Wt.%**	0.75	0.13	0.08	4.49	0.01	0.07	0.02	0.03	0.01	Balance

**Table 2:** FSSW process parameters show experimental matrix.

Experimental code	Plunge depth (mm)	Tilt angle (°)	Dwell time (s)	Rotational speed (rpm)
S1	0.2	2	5	600
S2	0.2	2	5	900
S3	0.2	2	5	1200
S4	0.2	2	10	600
S5	0.2	2	10	900
S6	0.2	2	10	1200
S7	0.2	2	15	600
S8	0.2	2	15	900
S9	0.2	2	15	1200

**Table 3:** The AISI H13 steel tool chemical composition.

**Element**	C	Si	Mo	Mn	V	Cr
**Wt. (%)**	0.40	1.00	1.35	0.40	1.00	5.25

**Table 4:** Tool design parameters used for AISI H13 steel tool.

Tool Design Parameters		
1	Tool Probe Profile (Shape), End Surface	tapered
2	Tool Probe Diameter (mm)	6
3	Tool Material	H13 Hot-working steel tool
4	Tool Probe Profile (Shape), Outer Surface	Cylindrical
5	Tool Shoulder Diameter (mm)	18
6	Tool Probe Length (mm)	6

**Table 5:** XRD Structural analysis measurement conditions.

Property	Specification
k	0.94
X-ray Current	30 mA
Scan range (2⊝)	Between 5 and 90°
Scan mode	Continues
X-ray Excitation voltage	40 kV
Filter	K-beta filter
Kα radiation	*ƛ* = 1.5406 Å
Scanning rate	1.0 deg/min (2⊝/seg)
Step width	0.01 deg
Incident Slit	2/3 deg
Kβ	*ƛ*=1.39225 Å
Detector	Scintillation counter

**Table 6:** XRD peak list for the base-metal- AA5083-H116.

No.	2-theta (deg)	d (ang.)	Height (cps)	FWHM (deg)	Int. I (cps deg)	Int. W (deg)	Size (ang.)
1	7.70	11.470	51	1.430	77	1.500	58
2	13.60	6.500	32	0.560	35	1.100	148
3	37.94	2.369	2609	0.194	595	0.228	452
4	44.19	2.047	1084	0.205	261	0.241	438
5	64.56	1.442	300	0.222	80.1	0.270	441
6	77.68	1.228	661	0.223	189.2	0.286	477
7	81.88	1.175	348	0.222	97.3	0.280	496

**Table 7:** XRD peak list for fabricated FSSW AA5083-H116 at 600 rpm, 15 s.

No.	2-theta (deg)	d (ang.)	Height (cps)	FWHM (deg)	Int. I (cps deg)	Int. W (deg)	Size (ang.)
1	8.18	10.800	168	2.440	436	2.600	34.0
2	20.10	4.420	11	2.600	33	2.900	32
3	38.02	2.365	4534	0.214	1117	0.246	411
4	44.28	2.044	1711	0.228	453	0.264	393
5	64.66	1.441	369	0.242	111.4	0.300	406
6	77.78	1.227	729	0.271	239	0.328	394
7	81.98	1.174	473	0.284	156.7	0.330	387

**Table 8:** XRD peak list for fabricated FSSW AA5083-H116 at 900 rpm, 15 s.

No.	2-theta (deg)	d (ang.)	Height (cps)	FWHM (deg)	Int. I (cps deg)	Int. W (deg)	Size (ang.)
1	7.91	11.170	67	0.990	70	1.000	84
2	9.46	9.340	71	1.280	96	1.400	65
3	13.69	6.460	29	0.850	26	0.900	98
4	16.48	5.380	15	1.500	24	1.600	57
5	34.29	2.6127	123	0.122	22.5	0.180	710
6	38.14	2.357	8574	0.178	1843	0.215	494
7	44.38	2.039	4366	0.172	955	0.219	520
8	64.75	1.439	1759	0.166	409	0.233	592
9	77.87	1.226	2252	0.184	581	0.258	580
10	82.07	1.173	670	0.191	175	0.262	577

**Table 9:** XRD peak list for fabricated FSSW AA5083-H116 at 1200 rpm, 15 s.

No.	2-theta (deg)	d (ang.)	Height (cps)	FWHM (deg)	Int. I (cps deg)	Int. W (deg)	Size (ang.)
1	8.43	10.480	65	1.870	130	2.000	44
2	13.70	6.460	35	0.850	45	1.300	99
3	16.54	5.360	26	0.660	24	0.900	127
4	34.14	2.624	72	0.213	17.7	0.250	407
5	38.02	2.365	6777	0.213	1728	0.255	412
6	44.28	2.044	3448	0.226	957	0.278	396
7	64.69	1.440	674	0.265	233	0.350	370
8	77.83	1.226	1305	0.293	491	0.376	364
9	82.05	1.174	618	0.281	223	0.360	392

**Table 10:** Average Vickers hardness data values at different zones of the spot-welds.

	BM (Hv)	HAZ (Hv)	TMAZ (Hv)	SZ (Hv)
S1 (600 rpm, 5 s)	98.52	100.36	104.31	108.32
S2 (900 rpm, 5 s)	99.33	102.2	108.01	112.36
S3 (1200 rpm, 5 s)	98.92	100.36	102.43	104.54
S4 (600 rpm, 10 s)	98.52	100.24	103.21	107.66
S5 (900 rpm, 10 s)	99.27	101.57	106.91	112.12
S6 (1200 rpm, 10 s)	98.63	100.15	102.41	105.22
S7 (600 rpm, 15 s)	98.73	99.70	102.58	106.80
S8 (900 rpm, 15 s)	99.13	101.97	106.95	112.62
S9 (1200 rpm, 15 s)	98.70	100.55	103.02	105.34

## 3 Experimental Design, Materials and Methods

This section documented various methods and the procedures used in carrying out friction stir spot welding (FSSW). Micro-hardness and XRD analysis employed for characterizing the welded samples of aluminium alloy 5083-H116 are explicitly described. The chemical composition of the parent material was also documented in this section. Brief introductions of the techniques used for characterization, equipment used, and the laboratory procedures for using the equipment are presented. The chemical compositions of the procured parent material AA5083-H116 are presented in Table 1 which conformed to the material safety data sheet (MSDS) of the supplier.

### 3.1 Methods

**Fig. 1:**
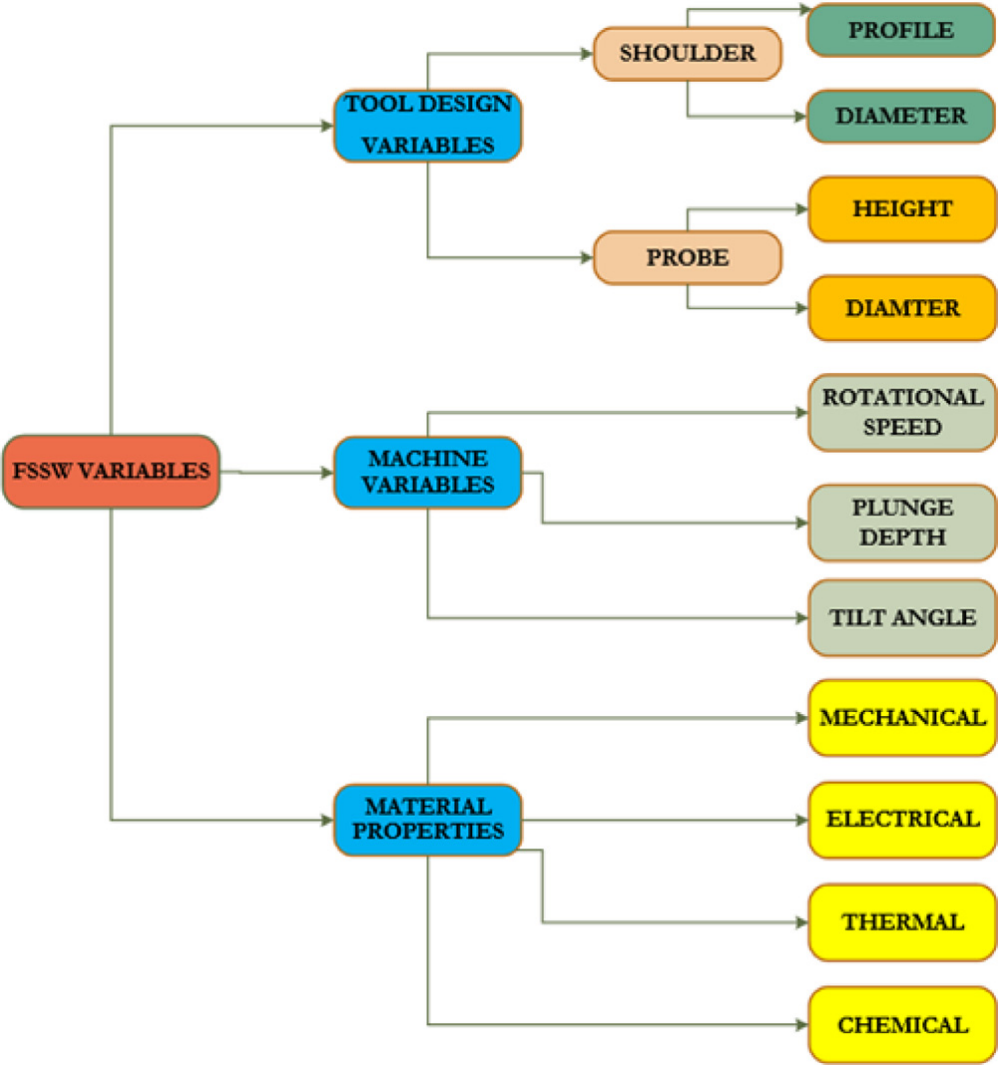
Schematic diagram of the variables used during FSSW.

**Fig. 2:**
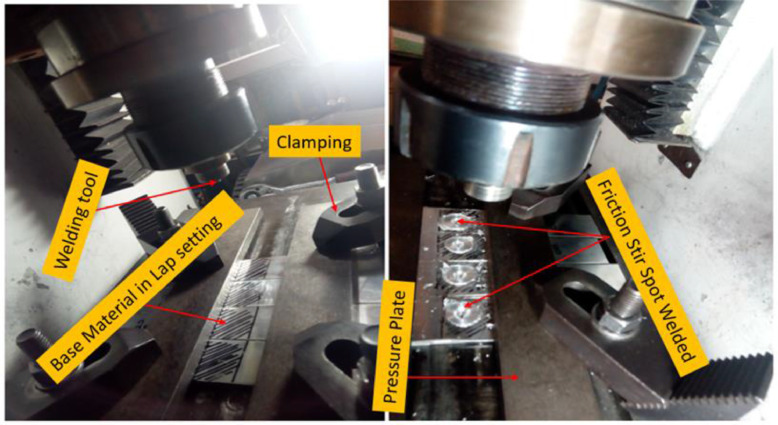
Snapshot of the Welding machine showing setting details and spot welds samples.

A 2T linear numerically control friction stir welding machine that was manufactured by ETA technology PVT Ltd, Bangalore, located at Indian Institute of Technology (IIT), Kharagpur, West Bengal, India was employed for the spot welds samples. The machine can store data during operation in which one will be able to retrieve at any point in time whenever the work is completed. Forces along X and Z direction can be recorded with the incorporation of a load cell into the machine [6,7]. The data were stored on the LabView software to monitor the activities of the welding in a real-time scenario. The machine tends to vary travel speed, rotational speed, tilt angles as well as plunge depth during operation. It must be noted that a controlled position was maintained during FSSW, the tool rotational speed used during the spot welding was in the step of 300 rpm from 600 rpm to 1200 rpm as shown in Table 2. There was no tool traverse movement and the dwell times were in the step of 5 s varying from 5 s to 15 s while the tool plunge rate was maintained although the experiment at 30 mm/min. The tool penetration depth (plunge) was noted to be 0.2 mm and the tool tilt angle was 2°. The process parameters used during this data acquisition is presented in Fig. 1. The illustration of the arrangement of the samples on the machine and the fabricated aluminium alloy samples are jointly presented in Fig. 2.

**Fig. 3:**
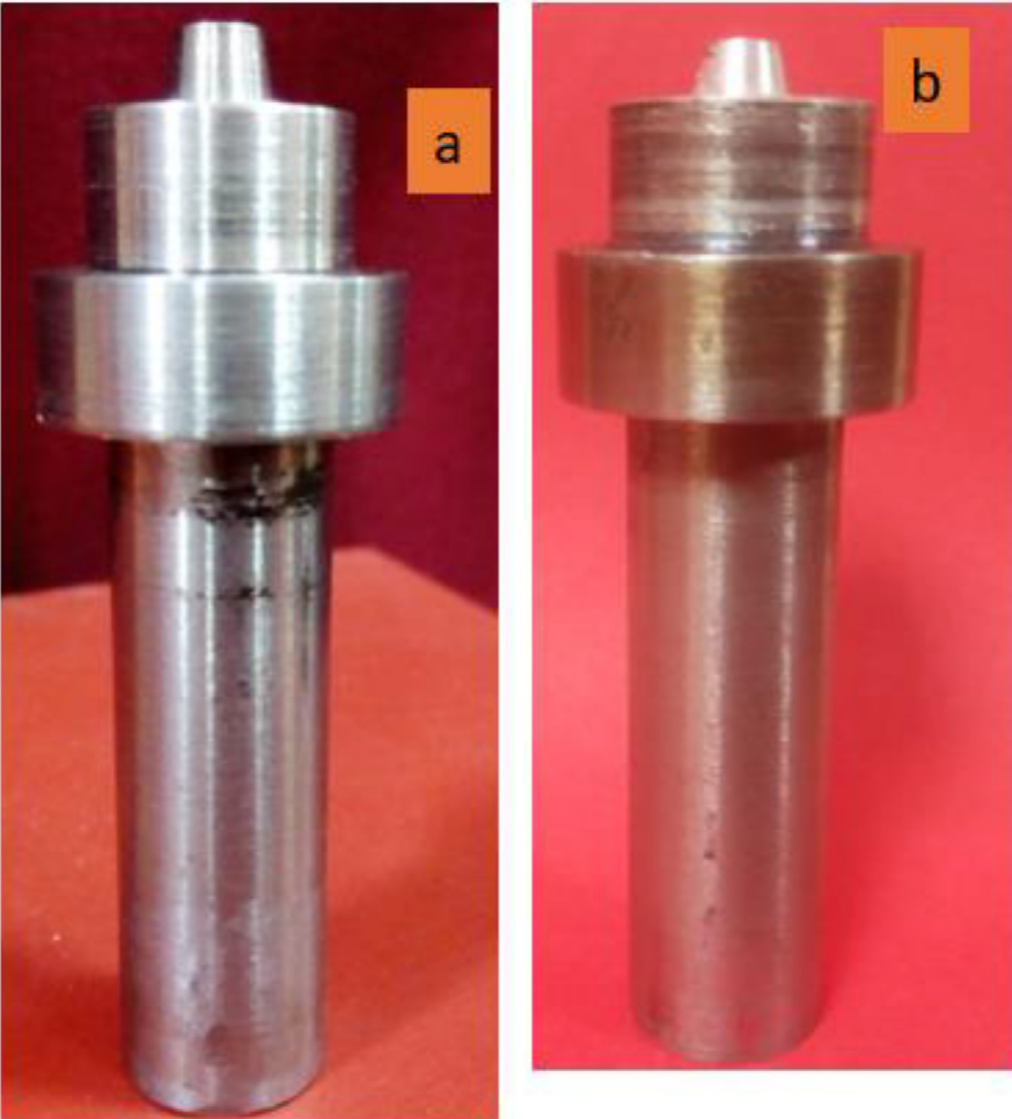
Fabricated AISI H13 welding Tool for Stirring and Mixing during FSSW (a) Newly produced (b) Used.

The welding tool was made from high strength AISI H13chrominium hot-work steel tool. The selection of this material to be used for the welding tool was based on the excellent properties it possessed such as a superb combination of fatigue resistance and hot toughness, excellent thermal shock resistance, easy to machine, high hardenability, and it can be well tolerated under water cooling condition. This material (AISI H13 steel tool) can be used as extrusion dies, inserts, pressure dies, hot forging dies, cores, casting tools, plastic mould, hot shear blades as well as stamping dies. In this dataset, a cylindrical tapered rotating tool was used with a probe length of 5 mm and probe diameter of 6 mm, it has a shoulder diameter of 18 mm which was initially 25 mm before machining. Fig. 3a shows the rotating tool design before use and Fig. 3b depicted the tool after used, the probe was taper at 10°. The probe was responsible for plunging and stirring while the shoulder was responsible to generate intense heat that will set the material into plastic deformation. The chemical composition of AISI H13 is presented in Table 3. The following mechanical properties propelled the choice of AISI H13 as welding tool over the other materials: ultimate tensile strength of 1990 MPa, machinability of 50%, elongation at break of 9.0%, the bulk modulus of 160 GPa, yield tensile strength of 1650 MPa, poisons ratio of 0.30, shear modulus of 81 GPa and modulus of elasticity of 210 GPa. The design of the rotating tool has the following parameters as shown in Table 4. The tool design has a shoulder diameter of 18 mm, the length of the probe, and the diameter of the probe was 6.0 mm each.

The microhardness examination was out carried using TH713 digital microhardness equipment with diamond indenter following ASTM E92–82 standard test method [8] standard. Microhardness values help to determine the resistance of the sample to plastic deformation, the strength of the sample, and the wear resistance of the sample. A controlled test force was employed to indent the samples. The indenter was pressed into the sample by an accurately controlled test force. The force of 100 g was maintained for a specific dwell time of 15 s. After the dwell time is completed, the indenter was removed leaving an indent on the sample that appears rhombic shaped on the surface. The size of the indent was determined optically by measuring the two diagonals of the square indent. Using the size of the indentation obtained, the hardness values of the sample were obtained. X-ray Diffraction (XRD) analysis was conducted on the parent material and one sample of the spot-welded material since there was no reinforcement used, this was done to determine the structural integrity, the mineralogical compositions and crystallized phases in the parent material as well as spot welded sample per the standard method, E112–12 [9]. The analysis was carried out using the Rigaku Ultima IV Multifunctional X-ray. The dataset was used to determine crystallite size of the parent material, line broadening (i.e. Full width at half maximum intensity (FWHM)) of the diffraction peak, The distance between planes of atoms that give rise to diffraction peaks which are called “d spacing “. The diffraction data (d value and relative intensity) obtained was compared to that of the standard data of minerals from the mineral powder diffraction file.

### 3.2 Experimental design

This section presented the data acquired from the microhardness testing of the spot-welded samples and structural integrity via XRD analysis to determine the efficacy of the friction stir spot welding (FSSW) on the AA5083. The data of X-ray diffraction (XRD) is presented for phases identification and to determine crystallite sizes in the welded zones and the parent materials.

The X-Ray diffractogram was characterized for the base-metal - (AA5083-H116) and the spot-welded ((AA5083-H116) materials for 600 rpm, 900 rpm, and 1200 rpm at 15 s. Each sample was run through the Rigaku Ultima IV Multifunctional X-ray, developed by the Rigaku International Corporation, Tokyo, Japan. The generator configuration was at 40 kV scan excitation voltage and 30 mA scan current. The scanning time was 1.0 deg/min (2⊝/seg) with a 0.01-degree step width. The scan distance (2⊝) was established between 5.0 and 90.0° having a steady k (constant) value of 0.94 and wavelength ƛ at 1.5406 Å. It was generated at a continuous value of CuKα radiation using incident split and interferometric counter-detectors and scan mode were continuous. To confirm the crystal structure and the mineralogical compositions of the samples used, XRD tests were conducted on both the base metal and fabricated spot-welded samples. The XRD was obtained with the help of an automatic divergence slice i.e. a sample length irradiated, regardless of the angle of Bragg (2θ) in degree. The formation and the distribution of crystallite size within the welded zone have been attributed to the following factors, vertical pressure, material chemistry, tool geometry, workpiece temperature, as well as significant active cooling. Grain development takes place at the end of recovery and recrystallization [10,11]. Table 5 shows the presentation of the measurement conditions under which the XRD analysis was conducted. The crystal structural phases, as well as diffraction patterns, were obtained as shown in Table 6, Table 7, Table 8, Table 9 for the base-metal (BM) as well as spot welded samples at 600 rpm, 900 rpm, and 1200 rpm respectively with their raw data in the attached Supplementary Materials (Raw data for Tables 6–8 respectively).

**Fig. 4:**
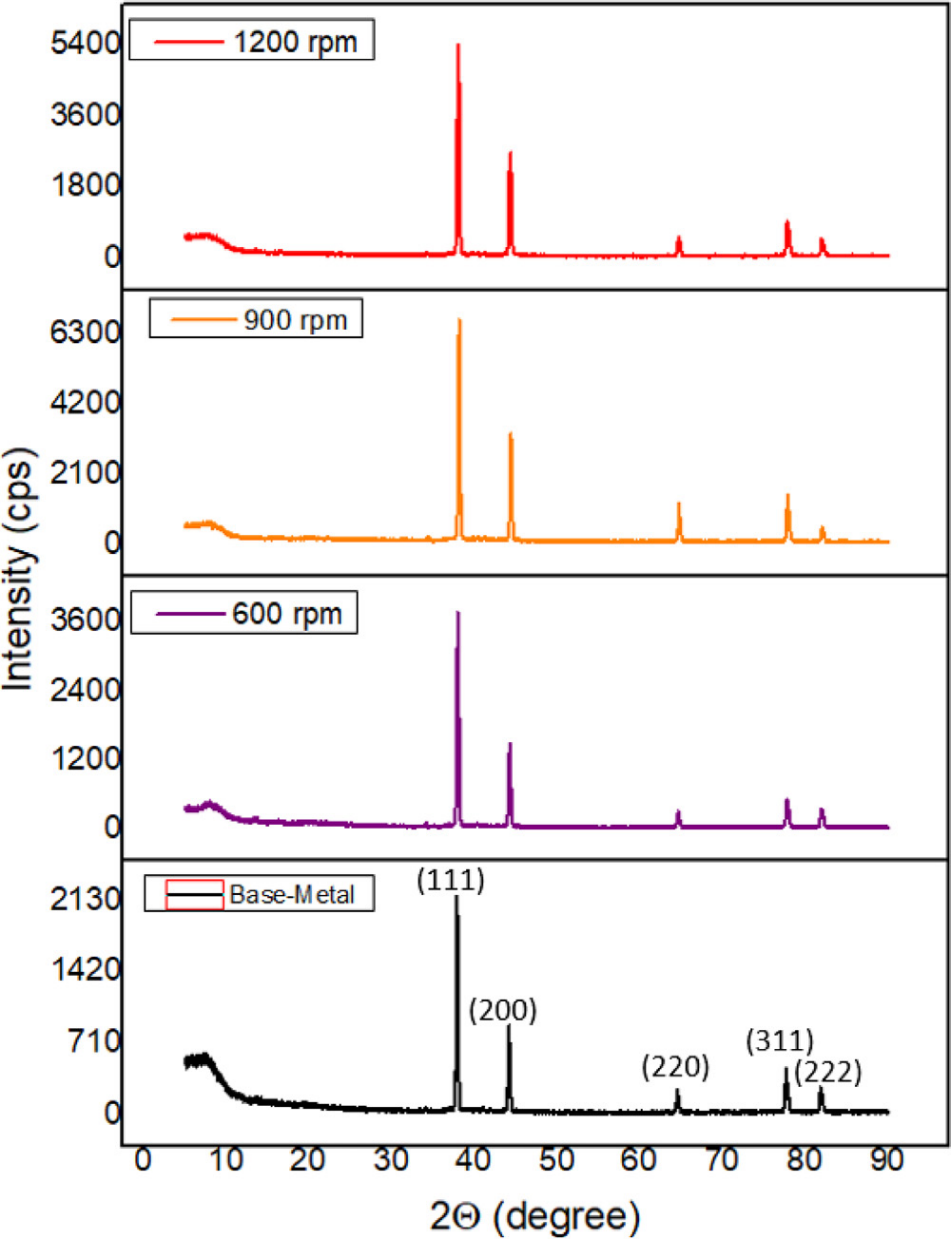
XRD Pattern for the base-metal and friction stir spot welds at 15 s.

Fig. 4 shows XRD spot welding diffractograms of AA5083-H116 specimens for the base-metal and friction stir spot welds at a different rotational speed of 600 rpm, 900 rpm, and 1200 rpm at a steady dwell time of 15 s, the raw data for plotting each peak intensity can be found in the attached Supplementary Materials (“Raw data for Fig. 4″). The crystallite sizes, FWHM - Full width at half maximum intensity of the diffraction peak, height, intensity values were all obtained from the machine data regarding standard data of International Centre for Diffraction Data (ICDD) with basic cross-reference (ICSD:41,447, ICDD:04–008–4821 [12,19]; ICSD:82,134, ICDD:04–003–2900 [13,20] and they are computed from POWD-12++ [13,20]. Fig. 4 shows the XRD pattern for the base-metal as well as the fabricated spot-welds.

**Fig. 5:**
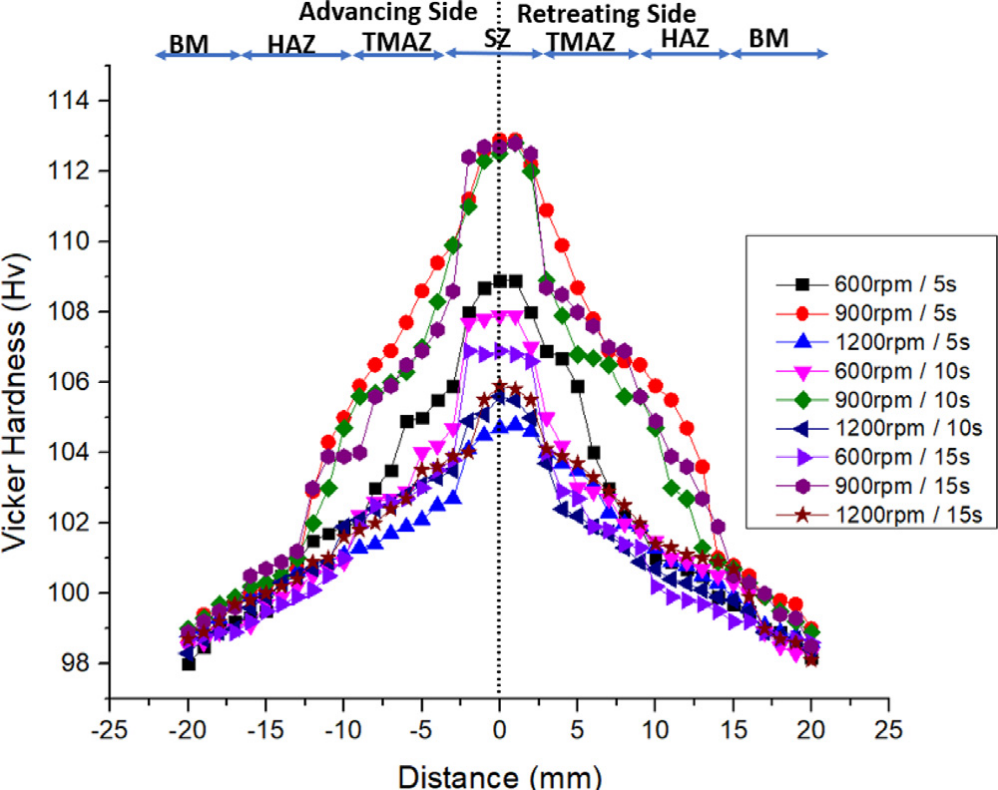
Microhardness data profile of the friction stir welded samples.

**Fig. 6:**
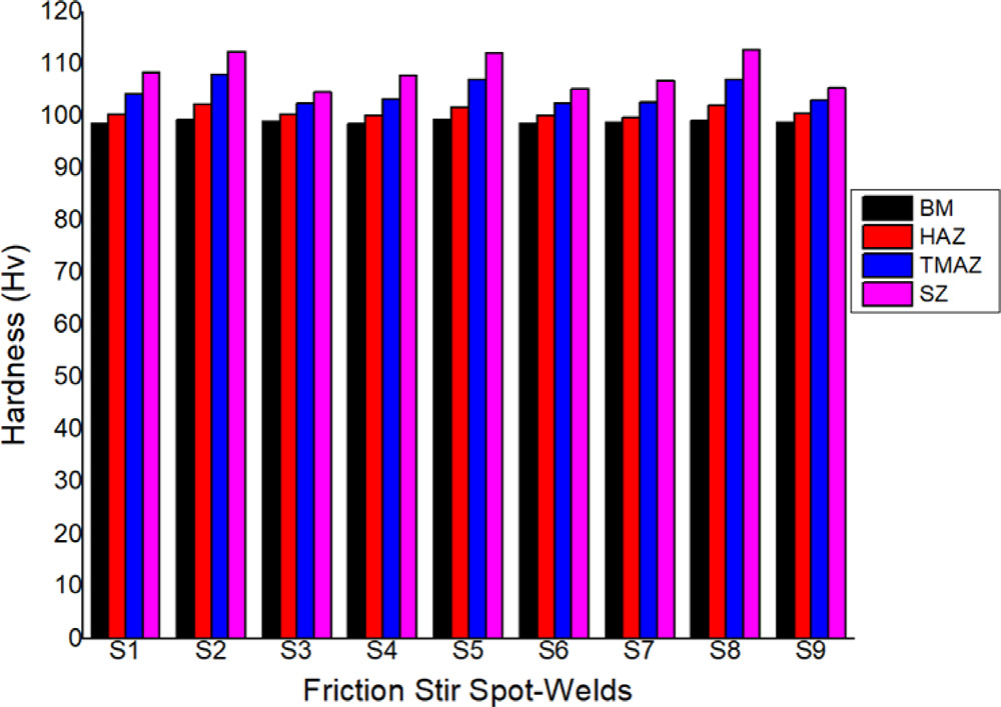
Plot of average Vickers hardness data values at different zones of the spot-welds.

The hardness values for the parent material and the spot-welded materials were obtained through Hv scale test equipment from Vickers Hardness. For the parent material of 4 mm AA 5083-H116 material, the average hardness values obtained by the Vickers hardness testing system was 98 Hv for a typical 4 mm AA 5083-H116 material which is the standard Vicker hardness for the base-metal AA5083. It must be established that among several other attributes, hardness is one of the mechanical characteristics that enables a material to withstand plastic deformation, penetration, as well as scratching [14,15]. It also helps to determine if the material treatment is sufficient for the intended reason [[16], [17], [18],^21^]. Fig. 5 shows the hardness profile of the spot-welds generated by FSSW. The raw data for plotting the microhardness profile in Fig. 5 can be found in the attached Supplementary Material (“Raw data for Fig. 5″). The data investigated SZ, HAZ, TMAZ, and BM for each sample at both advancing side (AS) and retreating side (RS) which is at the left and right direction of the FSSW joint. The highest hardness was obtained at the SZ of the welded area. The average hardness values at different zones are presented in Table 10 and Fig. 6.

## Ethics Statement

The data resulted from experimental neither on animal models nor with human volunteers.

## Declaration of Competing Interest

Authors declared that there is no known conflict of interest.

## Appendix Supplementary materials

Image, application 1

Image, application 2

Image, application 3

Image, application 4

Image, application 5

Image, application 6

Image, application 7

Image, application 8

Image, application 9

## References

[1] Tamasgavabari R., Reza A., Mehdi S., Reza A. (2018). The effect of harmonic vibration with a frequency below the resonant range on the mechanical properties of AA-5083-H321 aluminium alloy GMAW welded parts d b. Mater. Sci. Eng. A.

[2] Zhu C., Cheon J., Tang X., Na S., Cui H. (2018). International journal of heat and mass transfer molten pool behaviours and their influences on welding defects in narrow gap GMAW of 5083 Al-alloy. Int. J. Heat Mass Transf..

[3] Su Y., Hua X., Wu Y. (2013). Effect of input current modes on the intermetallic layer and mechanical property of aluminium-steel lap joint obtained by gas metal arc welding. Mater. Sci. Eng. A.

[4] Peasura P., Watanapa A. (2012). Influence of shielding gas on aluminum alloy 5083 in gas tungsten arc welding. Int. Workshop Inf. Electron. Eng..

[5] Atlas Steel. 2013. *Aluminium alloy data sheet 5083*. Available from: www.atlassteels.com.au.

[6] Ikumapayi O.M., Akinlabi E.T., Majumdar J.D., A.Akinlabi S. (2019). Influence of 17-4PH stainless steel and α+β titanium alloy powders for corrosion susceptibility on friction stir processed AA7075-T651 aluminium matrix composites. J. Bio Tribo Corros..

[7] Ikumapayi O.M., Akinlabi E.T. (2019). Efficacy of α-β grade titanium alloy powder (Ti-6Al-2Sn-2Zr-2Mo-2Cr-0.25Si) in surface modification and corrosion mitigation in 3.5% NaCl on friction stir processed armour grade 7075-T651 aluminium alloys – insight in defence applications. Mater. Res. Express.

[8] G. F. Vander Voort, Metallography: Principles and Practice, McGraw-Hill Book Co., NY, 1984; ASM International, Materials Park, OH, 1999, pp. 356, 357, 380–381

[9] Standard Test Methods for Determining Average Grain Size, E112-12. *Copyright ©ASM International, USA*. 2013.

[10] Ikumapayi O.M., Akinlabi E.T., Sharma A., Sharma V., Oladijo O.P. (2020). Tribological, structural and mechanical characteristics of friction stir processed aluminium-based matrix composites reinforced with stainless steel micro-particles. Eng. Solid Mech..

[11] Offor P.O., Okorie B.A., Ezema F.I., Aigbodion V.S., Daniel-mkpume C.C. (2015). Synthesis and characterization of nanocrystalline zinc sulphide thin films by chemical spray pyrolysis. J. Alloy. Compd..

[12] Huang J., He X., Guo Y., Zhang Z., Shi Y., Fan D. (2017). Joining of aluminium alloys to galvanized mild steel by the pulsed DE-GMAW with the alternation of droplet transfer. J. Manuf. Process..

[13] Sadykov V.A., Isupova L.A., Tsybulya S.V., Cherepanova S.V., Litvak G.S., Burgina E.B., Kustova G.N., Kolomiichuk V.N., Ivanov V.P., Paukshtis E.A., Golovin A.V., Avvakumov E.G. (1996). Effect of mechanical activation on the real structure and reactivity of iron (iii) oxide with corundum-type structure. J. Solid State Chem..

[14] Mulaba-Kapinga D., Nyembwe K.D., Ikumapayi O.M., Akinlabi E.T. (2020). Mechanical, electrochemical and structural characteristics of friction stir spot welds of aluminium alloy 6063. Manuf. Rev..

[15] Ikumapayi O.M., Akinlabi E.T., Abegunde O.O., Fayomi O.S.I. (2020). Electrochemical investigation of calcined agrowastes powders on friction stir processing of aluminium-based matrix composites. Mater. Today Proc..

[16] Trueba L., Heredia G., Rybicki D., Johannes L.B. (2015). Effect of tool shoulder features on defects and tensile properties of friction stir welded aluminium 6061-T6. J. Mater. Process. Technol..

[17] Jonckheere C., de Meester B., Cassiers C., Delhaye M., Simar A. (2012). Fracture and mechanical properties of friction stir spot welds in 60603 – T6 aluminium alloy. Int. J. Adv. Manuf. Technol..

[18] Ikumapayi O.M., Akinlabi E.T. (2019). Recent advances in keyhole defects repairs via refilling friction stir spot welding. Mater. Today Proc..

[19] Akinlabi E.T., Ikumapayi O.M., Bodunde O.P., Adaramola B.A., Uchegbu I.D., Fatoba S.O. (2020). Impact of quenching on the hardenability of steels EN-3 (∼1015), EN-8 (∼1040) and EN-24 (∼4340) during jominy end quench technique. Int. J. Emerging Technol..

[20] Afolalu S.A., Samuel O.D., Ikumapayi O.M. (2020). Development and characterization of nano- flux welding powder from calcined coconut shell ash admixture with FeO particles. J. Mater. Res. Technol..

[21] Afolalu S.A., Samuel O.D., Ikumapayi O.M., Oladipupo S., Emetere M.E. (2020). Study of mechanical behaviours and characterization of steel joints in mig welding under varying fluxes. Int. J. Eng. Res. Technol..

