# Supplementary material for: Data on microhardness and structural analysis of friction stir spot welded lap joints of AA5083-H116
Source: Data Brief. 2020 Nov 30;33:106585. doi: 10.1016/j.dib.2020.106585 (PMC8129645; doi:10.1016/j.dib.2020.106585)
Supplement: Supplementary file 6 [file mmc6.docx]

**Analysis Results**

**General Information**

| Analysis date | 2019/04/23 12:47:05 PM | | |
| --- | --- | --- | --- |
| Sample name | AA5083 (BM) | Measurement date | 2019/04/13 14:36:30 |
| File name | AA5083 (BM) | Operator | User |
| Comment |  | | |

**Measurement profile**

**Measurement conditions**

| X-Ray | 40 kV , 30 mA | Scan speed / Duration time | 1.0000 deg./min. |
| --- | --- | --- | --- |
| Goniometer |  | Step width | 0.0100 deg. |
| Attachment | - | Scan axis | 2theta/theta |
| Filter | K-beta filter | Scan range | 5.0000 - 90.0000 deg. |
| CBO selection slit | - | Incident slit | 2/3deg. |
| Diffrected beam mono. |  | Length limiting slit | - |
| Detector | Scintillation counter | Receiving slit #1 | 2/3deg. |
| Scan mode | CONTINUOUS | Receiving slit #2 | 0.60mm |

**Qualitative analysis results**

| Phase name | Formula | Figure of merit | Phase reg. detail | DB card number |
| --- | --- | --- | --- | --- |
| Aluminum, syn | Al | 0.603 | ICDD (PDF2010) | 01-072-3440 |
| Aluminum Iron Silicide | Al4 Fe1.7 Si | 1.906 | ICDD (PDF2010) | 01-075-6157 |

| Phase name | Formula | Space group | Phase reg. detail | DB card number |
| --- | --- | --- | --- | --- |
| Aluminum, syn | Al | 225 : Fm-3m | ICDD (PDF2010) | 01-072-3440 |
| Aluminum Iron Silicide | Al4 Fe1.7 Si | 194 : P63/mmc | ICDD (PDF2010) | 01-075-6157 |

**Peak list**

| No. | 2-theta(deg) | d(ang.) | Height(cps) | FWHM(deg) | Int. I(cps deg) | Int. W(deg) | Size(ang.) |
| --- | --- | --- | --- | --- | --- | --- | --- |
| 1 | 7.70(13) | 11.47(19) | 51(9) | 1.43(12) | 77(8) | 1.5(4) | 58(5) |
| 2 | 13.60(7) | 6.50(3) | 32(7) | 0.56(12) | 35(3) | 1.1(4) | 148(32) |
| 3 | 37.942(3) | 2.3695(2) | 2609(66) | 0.194(2) | 595(4) | 0.228(7) | 452(5) |
| 4 | 44.199(4) | 2.04744(17) | 1084(43) | 0.205(3) | 261(2) | 0.241(11) | 438(7) |
| 5 | 64.562(6) | 1.44228(13) | 300(22) | 0.222(7) | 80.1(12) | 0.27(2) | 441(13) |
| 6 | 77.681(4) | 1.22822(5) | 661(33) | 0.223(4) | 189.2(18) | 0.286(17) | 477(9) |
| 7 | 81.886(5) | 1.17545(6) | 348(24) | 0.222(6) | 97.3(14) | 0.28(2) | 496(12) |
