# Supplementary material for: Data on microhardness and structural analysis of friction stir spot welded lap joints of AA5083-H116
Source: Data Brief. 2020 Nov 30;33:106585. doi: 10.1016/j.dib.2020.106585 (PMC8129645; doi:10.1016/j.dib.2020.106585)
Supplement: Supplementary file 7 [file mmc7.docx]

**Analysis Results**

**General Information**

| Analysis date | 2019/04/23 12:48:49 PM | | |
| --- | --- | --- | --- |
| Sample name | AA5083 @ 600 RPM | Measurement date | 2019/04/13 17:33:58 |
| File name | AA5083 @ 600 RPM | Operator | User |
| Comment |  | | |

**Measurement profile**

**Measurement conditions**

| X-Ray | 40 kV , 30 mA | Scan speed / Duration time | 1.0000 deg./min. |
| --- | --- | --- | --- |
| Goniometer |  | Step width | 0.0100 deg. |
| Attachment | - | Scan axis | 2theta/theta |
| Filter | K-beta filter | Scan range | 5.0000 - 90.0000 deg. |
| CBO selection slit | - | Incident slit | 2/3deg. |
| Diffrected beam mono. |  | Length limiting slit | - |
| Detector | Scintillation counter | Receiving slit #1 | 2/3deg. |
| Scan mode | CONTINUOUS | Receiving slit #2 | 0.60mm |

**Qualitative analysis results**

| Phase name | Formula | Figure of merit | Phase reg. detail | DB card number |
| --- | --- | --- | --- | --- |
| Aluminum | Al | 0.637 | ICDD (PDF2010) | 01-073-2661 |
| Iron Titanium | Fe2 Ti | 2.629 | ICDD (PDF2010) | 03-065-3571 |

| Phase name | Formula | Space group | Phase reg. detail | DB card number |
| --- | --- | --- | --- | --- |
| Aluminum | Al | 225 : Fm-3m | ICDD (PDF2010) | 01-073-2661 |
| Iron Titanium | Fe2 Ti | 194 : P63/mmc | ICDD (PDF2010) | 03-065-3571 |

**Peak list**

| No. | 2-theta(deg) | d(ang.) | Height(cps) | FWHM(deg) | Int. I(cps deg) | Int. W(deg) | Size(ang.) |
| --- | --- | --- | --- | --- | --- | --- | --- |
| 1 | 8.18(5) | 10.80(6) | 168(17) | 2.44(4) | 436(11) | 2.6(3) | 34.0(6) |
| 2 | 20.1(3) | 4.42(7) | 11(4) | 2.6(3) | 33(5) | 2.9(16) | 32(4) |
| 3 | 38.016(2) | 2.36502(14) | 4534(87) | 0.2136(15) | 1117(5) | 0.246(6) | 411(3) |
| 4 | 44.280(4) | 2.04386(16) | 1711(53) | 0.228(3) | 453(3) | 0.264(10) | 393(5) |
| 5 | 64.661(6) | 1.44030(12) | 369(25) | 0.242(7) | 111.4(14) | 0.30(2) | 406(11) |
| 6 | 77.778(5) | 1.22693(6) | 729(35) | 0.271(5) | 239(2) | 0.328(19) | 394(7) |
| 7 | 81.978(6) | 1.17436(7) | 473(28) | 0.284(6) | 156.7(18) | 0.33(2) | 387(8) |
