# Supplementary material for: Data on microhardness and structural analysis of friction stir spot welded lap joints of AA5083-H116
Source: Data Brief. 2020 Nov 30;33:106585. doi: 10.1016/j.dib.2020.106585 (PMC8129645; doi:10.1016/j.dib.2020.106585)
Supplement: Supplementary file 8 [file mmc8.docx]

**Analysis Results**

**General Information**

| Analysis date | 2019/04/23 12:49:58 PM | | |
| --- | --- | --- | --- |
| Sample name | AA5083 @ 900RPM | Measurement date | 2019/04/15 06:20:29 |
| File name | AA5083 @ 900RPM | Operator | User |
| Comment |  | | |

**Measurement profile**

**Measurement conditions**

| X-Ray | 40 kV , 30 mA | Scan speed / Duration time | 1.0000 deg./min. |
| --- | --- | --- | --- |
| Goniometer |  | Step width | 0.0100 deg. |
| Attachment | - | Scan axis | 2theta/theta |
| Filter | K-beta filter | Scan range | 5.0000 - 90.0000 deg. |
| CBO selection slit | - | Incident slit | 2/3deg. |
| Diffrected beam mono. |  | Length limiting slit | - |
| Detector | Scintillation counter | Receiving slit #1 | 2/3deg. |
| Scan mode | CONTINUOUS | Receiving slit #2 | 0.60mm |

**Qualitative analysis results**

| Phase name | Formula | Figure of merit | Phase reg. detail | DB card number |
| --- | --- | --- | --- | --- |
| Aluminum | Al | 0.294 | ICDD (PDF2010) | 01-073-2661 |
| beta-Al5.15 Mg3.15 | Al5.15 Mg3.15 | 1.187 | ICDD (PDF2010) | 01-073-2626 |

| Phase name | Formula | Space group | Phase reg. detail | DB card number |
| --- | --- | --- | --- | --- |
| Aluminum | Al | 225 : Fm-3m | ICDD (PDF2010) | 01-073-2661 |
| beta-Al5.15 Mg3.15 | Al5.15 Mg3.15 | 221 : Pm-3m | ICDD (PDF2010) | 01-073-2626 |

**Peak list**

| No. | 2-theta(deg) | d(ang.) | Height(cps) | FWHM(deg) | Int. I(cps deg) | Int. W(deg) | Size(ang.) |
| --- | --- | --- | --- | --- | --- | --- | --- |
| 1 | 7.91(10) | 11.17(14) | 67(11) | 0.99(9) | 70(145) | 1(2) | 84(8) |
| 2 | 9.46(4) | 9.34(3) | 71(11) | 1.28(10) | 96(11) | 1.4(4) | 65(5) |
| 3 | 13.69(10) | 6.46(4) | 29(7) | 0.85(10) | 26(2) | 0.9(3) | 98(11) |
| 4 | 16.48(8) | 5.38(2) | 15(5) | 1.5(2) | 24(4) | 1.6(8) | 57(8) |
| 5 | 34.294(9) | 2.6127(7) | 123(14) | 0.122(17) | 22.5(15) | 0.18(3) | 710(97) |
| 6 | 38.144(2) | 2.35735(14) | 8574(120) | 0.1776(17) | 1843(9) | 0.215(4) | 494(5) |
| 7 | 44.382(3) | 2.03942(12) | 4366(85) | 0.172(2) | 955(6) | 0.219(6) | 520(7) |
| 8 | 64.748(2) | 1.43859(5) | 1759(54) | 0.166(3) | 409(3) | 0.233(9) | 592(9) |
| 9 | 77.865(3) | 1.22577(3) | 2252(61) | 0.184(2) | 581(3) | 0.258(9) | 580(7) |
| 10 | 82.070(5) | 1.17328(6) | 670(33) | 0.191(4) | 175(2) | 0.262(16) | 577(13) |
