# Supplementary material for: Data on microhardness and structural analysis of friction stir spot welded lap joints of AA5083-H116
Source: Data Brief. 2020 Nov 30;33:106585. doi: 10.1016/j.dib.2020.106585 (PMC8129645; doi:10.1016/j.dib.2020.106585)
Supplement: Supplementary file 9 [file mmc9.docx]

**Analysis Results**

**General Information**

| Analysis date | 2019/04/23 12:44:35 PM | | |
| --- | --- | --- | --- |
| Sample name | AA5083 @ 1200 rpm | Measurement date | 2019/04/15 17:37:11 |
| File name | AA5083 @ 1200 rpm | Operator | User |
| Comment |  | | |

**Measurement profile**

**Measurement conditions**

| X-Ray | 40 kV , 30 mA | Scan speed / Duration time | 1.0000 deg./min. |
| --- | --- | --- | --- |
| Goniometer |  | Step width | 0.0100 deg. |
| Attachment | - | Scan axis | 2theta/theta |
| Filter | K-beta filter | Scan range | 5.0000 - 90.0000 deg. |
| CBO selection slit | - | Incident slit | 2/3deg. |
| Diffrected beam mono. |  | Length limiting slit | - |
| Detector | Scintillation counter | Receiving slit #1 | 2/3deg. |
| Scan mode | CONTINUOUS | Receiving slit #2 | 0.60mm |

**Qualitative analysis results**

| Phase name | Formula | Figure of merit | Phase reg. detail | DB card number |
| --- | --- | --- | --- | --- |
| Aluminum | Al | 0.491 | ICDD (PDF2010) | 01-073-2661 |
| Iron Silicon | Fe Si2 | 2.919 | ICDD (PDF2010) | 03-065-2795 |

| Phase name | Formula | Space group | Phase reg. detail | DB card number |
| --- | --- | --- | --- | --- |
| Aluminum | Al | 225 : Fm-3m | ICDD (PDF2010) | 01-073-2661 |
| Iron Silicon | Fe Si2 | 123 : P4/mmm | ICDD (PDF2010) | 03-065-2795 |

**Peak list**

| No. | 2-theta(deg) | d(ang.) | Height(cps) | FWHM(deg) | Int. I(cps deg) | Int. W(deg) | Size(ang.) |
| --- | --- | --- | --- | --- | --- | --- | --- |
| 1 | 8.43(13) | 10.48(16) | 65(10) | 1.87(11) | 130(11) | 2.0(5) | 44(3) |
| 2 | 13.70(7) | 6.46(3) | 35(8) | 0.85(10) | 45(5) | 1.3(4) | 99(11) |
| 3 | 16.54(7) | 5.36(2) | 26(7) | 0.66(10) | 24(3) | 0.9(4) | 127(19) |
| 4 | 34.138(13) | 2.6242(10) | 72(11) | 0.213(18) | 17.7(14) | 0.25(6) | 407(34) |
| 5 | 38.023(2) | 2.36456(12) | 6777(106) | 0.2132(15) | 1728(7) | 0.255(5) | 412(3) |
| 6 | 44.276(2) | 2.04405(11) | 3448(76) | 0.2261(19) | 957(4) | 0.278(7) | 396(3) |
| 7 | 64.688(6) | 1.43977(12) | 674(34) | 0.265(6) | 233(2) | 0.35(2) | 370(9) |
| 8 | 77.825(4) | 1.22630(5) | 1305(47) | 0.293(4) | 491(3) | 0.376(16) | 364(5) |
| 9 | 82.050(6) | 1.17351(7) | 618(32) | 0.281(7) | 223(2) | 0.36(2) | 392(9) |
